# A New 2*α*,5*α*,10*β*,14*β*-tetraacetoxy-4(20),11-taxadiene (SIA) Derivative Overcomes Paclitaxel Resistance by Inhibiting MAPK Signaling and Increasing Paclitaxel Accumulation in Breast Cancer Cells

**DOI:** 10.1371/journal.pone.0104317

**Published:** 2014-08-05

**Authors:** Mei Mei, Dan Xie, Yi Zhang, Jing Jin, Feng You, Yan Li, Jungui Dai, Xiaoguang Chen

**Affiliations:** State Key Laboratory of Bioactive Substance and Function of Natural Medicines, Institute of Materia Medica, Chinese Academy of Medical Sciences and Peking Union Medical College, Beijing City, China; National Cancer Center, Japan

## Abstract

Tumor resistance due to multiple mechanisms seriously hinders the efficacy of chemotherapy drugs such as paclitaxel. The most widely studied P-glycoprotein inhibitors still have limited ability to reverse resistance in the clinic. In this study, NPB304, a novel Sinenxan A (SIA) derivative, was found to significantly sensitize resistant breast cancer cells to paclitaxel *in vitro* and *in vivo*. Treatment with NPB304 increased paclitaxel-induced apoptosis in a p53-dependent manner through PARP cleavage. Importantly, NPB304 enhanced the antitumor effect of paclitaxel in resistant breast tumor xenografts in nude mice without significantly affecting weight loss. NPB304 regulated cell resistance through inhibition of MAPK pathway components, including p-ERK and p-p38. Moreover, NPB304 increased paclitaxel accumulation by affecting P-gp function. In addition to increasing Rhodamine 123 accumulation, NPB304 promoted bidirectional permeability but decreased the efflux ratio of paclitaxel in a Caco-2 monolayer model, thereby increasing the intracellular concentration of paclitaxel. Similarly, NPB304 increased the concentration of paclitaxel in the resistant tumor tissue. Hence, NPB304 is a novel compound that promotes the sensitization of resistant cells to paclitaxel through multiple mechanisms and has the potential for use in combination therapies to treat resistant breast cancer.

## Introduction

Paclitaxel is a first-line therapeutic agent used clinically to treat breast cancer, ovarian cancer, non-small cell lung cancer and many other human malignancies [1]. Paclitaxel mainly exerts its anti-cancer effects by stabilizing microtubules, resulting in cell cycle arrest and apoptotic death of cancer cells [2], [3].

Although great progress and breakthroughs have been made, tumor resistance is a primary obstacle to the use of paclitaxel and remains an unsolved problem in the clinic [4]. Multidrug resistance (MDR) may involve multiple mechanisms [5], [6], including activation of the MAPK pathway [7], [8] and overexpression of P-glycoprotein (P-gp) [9], [10].

The MAPK pathway is activated by a variety of stimuli, including growth factors, which regulate cell survival, growth and differentiation. Previous studies have shown that chemotherapy drugs activate the MAPK pathway [11], [12], leading to resistance in cancer cells [7]. For example, the MAPK pathway can modulate anti-apoptotic molecules that are associated with drug resistance, whereas a combination of TKIs [13], [14] and MEK inhibitors [15], [16] can potentiate the efficacy of the drugs.

P-gp, an ATP-dependent efflux pump, can decrease the intracellular concentration of paclitaxel and weaken its anti-neoplastic effect [17]. Clinical studies of breast cancer have indicated that tumors expressing MDR-1 or P-gp show a decreased response to chemotherapy [18]. However, adverse reactions and side effects have limited the clinical use of P-gp inhibitors [19], [20].

**Figure 1:**
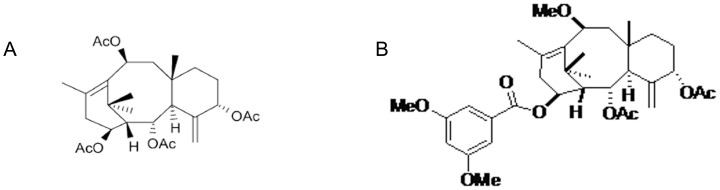
The chemical structures of SIA (A) and NPB304 (B).

Sinenxan A (2*α*,5*α*,10*β*,14*β*–tetraacetoxy-4(20), 11-taxadiene, SIA) (Fig. 1A) was isolated from callus cultures of *Taxus yunnanensis*. The literature has suggested that SIA, which has a taxane skeleton, could be used for the semi-synthesis of taxanes. In previous studies, SIA analogs or derivatives exhibited the potential to overcome MDR in resistant cancer cells, including MX-1/paclitaxel cells, A549/paclitaxel cells and KB/Vcr cells [21]–[24]. Therefore, further optimization of SIA to obtain more derivatives with improved structures and activity with multiple modes of action was warranted. NPB304 (2α,5α-diacetoxy-14β-3,5-dimethoxybenzoyl-10β-methoxy-taxa-4(20),11-diene) (Fig. 1B) was selected by screening a library of SIA derivatives, on the basis of its appropriate structure, outstanding activity, low cytotoxicity and multiple modes of action.

Here, we report that NPB304 overcomes paclitaxel resistance in breast cancer cells by inhibiting the MAPK pathway and increasing paclitaxel accumulation owing to its effects on P-gp function. Importantly, we also found that NPB304 was capable of increasing the antitumor efficacy of paclitaxel in paclitaxel-resistant breast tumor xenografts.

## Materials and Methods

### Reagents

NPB304, a biosynthetic taxane, is a white powder with the molecular formula C_34_H_46_O_9_ and a molecular weight of 598 g/mol; its chemical structure is shown in Fig. 1B. Paclitaxel, verapamil (Vrp), 1-(4,5-dimethylthiazol-2-yl)-3,5-diphenyformazan (MTT), 4′,6-diamidino-2-phenylindole (DAPI) and other chemicals were purchased from Sigma-Aldrich Corporation (USA). P-gp and actin antibodies were purchased from Santa Cruz Biotechnology (USA). Other antibodies, U0126 and 12-O-tetradecanoyl-phorbol-13-acetate (TPA/PMA) were purchased from Cell Signaling Technology, Inc.

### Cell lines and culture

The human breast cancer cell line MX-1 and its resistant derivative MX-1/paclitaxel [25] were generously provided by Professor Yongkui Jing (Mount Sinai School of Medicine, New York, USA) [26], [27]. The human breast cancer cell line MCF-7 was purchased from ATCC. MCF-7/paclitaxel was established by continuous stepwise exposure to paclitaxel [27]. The cells were cultured in DMEM (Gibco BRL) supplemented with 10% FBS at 37°C in a humidified atmosphere with 5% CO_2_. The indicated concentration of paclitaxel was added to the culture medium to maintain drug resistance. The resistant cells were grown in drug-free culture medium for more than 10 days before use in an assay. The human colon carcinoma cell line Caco-2 was purchased from the American Type Culture Collection (ATCC), and cells at passage numbers 25–35 were used for the assays.

### Cell cytotoxicity and colony formation assay

MTT assays were carried out as described previously [23]. Briefly, after 72 h of incubation, 100 µl of 0.5 mg/ml MTT (Sigma) was added and incubated for 4 h. Then, MTT was dissolved in DMSO, and the absorbance was measured at 570 nm using a microplate reader. For the colony formation assays, MCF-7/paclitaxel cells (200 cells/well) in 6-well plates were treated with paclitaxel alone or combined with NPB304 for 12 days. Subsequently, the cells were fixed with anhydrous methanol for 10 min and washed with PBS, and the colony numbers were counted after Giemsa staining (Beijing Cellchip Biotechnology Co., Ltd., Beijing, China) for 20 min.

### Annexin V-FITC/PI binding analysis

For the annexin V-FITC/PI assay, MCF-7/paclitaxel cells were collected after a 72-h treatment and stained with FITC-labeled annexin V and PI according to the manufacturer's instructions (KeyGEN Biotech Inc., China). The fluorescence levels were analyzed by flow cytometry (Beckman Coulter, USA).

### Immunofluorescence and confocal microscopy

MCF-7/paclitaxel cells were seeded onto glass coverslips in 24-well plates. After a 72-h treatment, the cells were fixed in 4% formaldehyde in PBS (pH 7.2–7.4) for 10 min at room temperature and permeabilized with Triton X-100 (0.5%, v/v) before blocking in 5% goat serum. Then, the cells were incubated with an anti-α-tubulin (1∶500) antibody overnight at 4°C. After washing three times in PBS, the cells were incubated with FITC-labeled secondary antibodies (1∶100) for 1 h and co-stained with DAPI. Then, the coverslips were mounted with antifade reagent (Beyotime, China) and observed using a Leica SP2 confocal microscope. The images were processed and analyzed using LAS-AF-Lite 2.6.0 software.

### Western blot analysis

Paclitaxel-resistant cells were lysed in RIPA buffer (50 mM Tris (pH 7.4), 150 mM NaCl, 1% Triton X-100, 1% sodium deoxycholate, 0.1% SDS, sodium orthovanadate, sodium fluoride and EDTA) containing protease inhibitor cocktails (Amresco, Solon, OH, USA). Equal amounts of cell lysates were resolved by SDS-PAGE and subsequently electrophoretically transferred to PVDF membranes (Millipore, Darmstadt, Germany). After blocking in TBST (10 mM Tris-HCl, 150 mM NaCl and 0.1% Tween 20, pH 8.0) with 5% non-fat milk (BD Biosciences, Franklin Lakes, NJ, USA) for 2 h at room temperature, the membranes were incubated with primary and secondary antibodies and subsequently visualized with an enhanced chemiluminescence detection kit (APPLYGEN Technologies Inc., Beijing, China). Actin was used as a loading control.

### Ethics Statement

The animal experiments were performed following a protocol approved by the Animal Ethics Committees of the Institute of Materia Medica, CAMS and PUMC. Full details of the study have been approved by the Animal Ethics Committee with permit number 00000011. The female BALB/c Nu mice (6–8 weeks) were provided free access to food and water.

### Xenograft models

A paclitaxel-resistant MX-1 xenograft model [27] was employed. Tumor resistance was achieved in the flank of female nude mice after eight passages (30 mg/kg paclitaxel per passage). Briefly, MX-1/paclitaxel tumor cells obtained from this model were grown to passages 9–12 and implanted subcutaneously into nude mice. The percentage of paclitaxel-resistant cells in passages 9–12 was in the range of 0–20%. When the tumors reached a volume of approximately 100 to 200 mm^3^ (day 5), the mice were randomized into treatment groups comprising six animals each. NPB304 was administered by oral gavage, and paclitaxel was administered by intravenous injection. NPB304 was suspended in a 0.5% solution of carboxymethyl cellulose. The tumor volumes and body weights of the mice were measured every 1 or 2 days. Tumor volume (V) was calculated using the equation V = 1/2×L×W^2^, and antitumor activity was expressed as relative volume (RTV). The mice were sacrificed after 15 days, and the tumors were removed, weighed and photographed. We harvested 4 tumors of equal weight from nude mice in the paclitaxel and combination groups to determine the intratumoral paclitaxel concentration using liquid chromatography-tandem mass spectrometry (LC-MS/MS) analysis and for Western blot assays.

### Rhodamine 123 (Rh123) accumulation

The effect of NPB304 on Rh123 accumulation was measured by flow cytometry analysis. Briefly, after treatment with or without NPB304 for 3 h, paclitaxel-resistant breast cancer cells were incubated with 10 µM Rh123 for an additional 0.5 h. Subsequently, the cells were harvested and washed three times prior to analysis by flow cytometry (Beckman Coulter, USA). The relative value was the ratio of the mean fluorescence intensity of intracellular Rh123 of the groups and that of the negative controls.

### Bidirectional permeability assay and determination of the intracellular concentration of paclitaxel in a Caco-2 monolayer model

Caco-2 cells (30,000 cells/insert) were seeded on Millicell cell culture insert polycarbonate filters (0.6 cm^2^) (Millipore, USA), which were incubated in a 24-well plate (Corning, USA) for 21 days. The culture medium was replaced every 2 days. The transepithelial electrical resistance (TEER) values of the cell monolayer were measured, and those that reached more than 400 ohms·cm^2^ were used in the assay. After being washed twice with warm HBSS buffer, the monolayers were balanced in HBSS buffer at 37°C for 30 min. The solutions containing the compounds were added to the apical side (for transport from the apical to the basolateral side; AP to BL) or the basolateral side (for transport from the basolateral side to the apical side; BL to AP), and drug-free HBSS was added to the opposite side. Samples were collected from the opposite side after 3 h. Subsequently, the Caco-2 cells were harvested and sonicated. The paclitaxel concentration was analyzed by LC-MS/MS as previously described [28].

### LC-MS/MS assay

The LC-MS/MS (Thermo Finnigan, USA) system was composed of a TSQ Quantum Access triple quadrupole mass spectrometer, a quaternary pump, a Surveyor autosampler, an automatic solvent degasser and electrospray ionization (ESI). The ESI conditions were optimized as follows: ion spray voltage, 4000 V; temperature, 300°C; sheath gas and auxiliary gas, nitrogen; pressure, 30 psi; and flow rate, 10 L/min. The chromatographic separation was achieved using a Zorbax C18 column (2.1×100 mm, 3.5 µm) with an online filter (0.5 µM, Upchurch Scientific Ltd.). The mobile phase was a mix of solvent A (0.1% formic acid in water) and B (0.1% formic acid in acetonitrile).

The assay was performed in positive electrospray ionization mode and detected in selected reactions monitoring (SRM) mode. The transitions were monitored at *m/z* 876.2→307.9 for paclitaxel. The data acquisition and analysis were automatically completed using Xcalibur 1.4.2 software.

### Statistical analysis

All the experiments were repeated 3 times, and the data are shown as the mean ± SD unless otherwise stated. Statistical analysis of the results was performed using a one-way ANOVA (with SPSS 16.0) or a t-test. p<0.05 was considered statistically significant.

## Results

### Synthesis of NPB304

We synthesized multiple SIA derivatives because they were previously found to be capable of overcoming drug resistance [21]–[24]. Three potent compounds were selected by MTT assay for preliminary *in vivo* experiments, and NPB304 was found to be the most effective.

NPB304 (Fig. 1B) was obtained by esterification using 2α,5α-diacetoxy-14β-hydroxy-10β-methoxy-taxa-4(20),11-diene as a starting material via a classic Knoevenagel condensation reaction with 3,5-dimethoxybenzoic acid. The reaction was carried out in anhydrous dichloromethane (CH_2_Cl_2_) in the presence of 1-(3-dimethyl-aminopropyl)-3-ethylcarbodiimide hydrochloride (EDCl) and 4-dimethylaminopyridine (DMAP) at room temperature under nitrogen. The corresponding mono-substituted products were obtained with an approximately 95% yield. The structure of NPB304 was identified by physical and chemical data collected by multiple analyses, such as HRMS and ^1^H NMR.

^1^H NMR (CDCl_3_, 300 MHz) *δ* ppm: 2.08 (s, 1H, H-1), 5.41 (br d, 1H, *J* = 5.7, H-2), 3.00 (d, 1H, *J* = 5.7, H-3), 5.28 (m, 1H, H-5), 1.90-1.70 (m, 2H, H-6a, b), 1.22 (m, 2H, H-7a, b), 2.32 (dd, 1H, *J* = 12.0, 15.0, H-9a), 1.90-1.70 (m, 1H, H-9b), 4.63 (dd, 1H, *J* = 5.4, 12.0, H-10), 2.94 (dd, 1H, *J* = 9.0, 19.2, H-13a), 2.56 (m, 1H, H-13b), 5.20 (m, 1H, H-14), 1.69 (s, 3H, 16-CH_3_), 1.31 (s, 3H, 17-CH_3_), 2.04 (s, 3H, 18-CH_3_), 0.86 (s, 3H, 19-CH_3_), 5.32 (m, 1H, H-20a), 4.89 (s, 1H, H-20b), 6.66 (s, 1H, H-25), 7.15 (s, 2H, H-23, 27), 2.02 (s, 3H, COCH_3_), 2.23 (s, 3H, COCH_3_), 3.31 (s, 3H, 10-OCH_3_), 3.83 (s, 6H, 24, 26-OCH_3_). HR-ESI-MS *m*/*z*: 621.3035 [M+Na]^+^ (calcd for C_34_H_46_O_9_Na 621.3039).

The positive HR-ESI-MS spectrum of NPB304 showed a quasi-molecular ion peak at *m/z* 621.3035 [M+Na]^+^, suggesting the molecular formula to be C_34_H_46_O_9_. The ^1^H NMR spectrum of NPB304 exhibited the signals of two methyl signals of acetyl moieties (*δ*_H_ 2.02, 2.23) together with four quaternary methyls (*δ* 1.69, 1.31, 2.04, 0.86), four oxygenated methylenes (*δ* 5.41, br d, 1H, *J* = 5.7, H-2; *δ* 4.63, dd, *J* = 12.0, 5.4 Hz, H-10; *δ* 5.28, s, H-5; *δ* 5.20, m, H-14), exocyclic methylene function protons (*δ* 5.32 and 4.89, br s, H-20), and a 3,5-dimethoxybenzoyl group (*δ* 6.66, s, 1H, H-25; *δ* 7.15, s, 2H, H-23, 27; *δ* 3.83, s, 6H, 24, 26-OCH_3_).

### NPB304 increases the sensitivity of resistant breast cancer cells to paclitaxel

**Figure 2:**
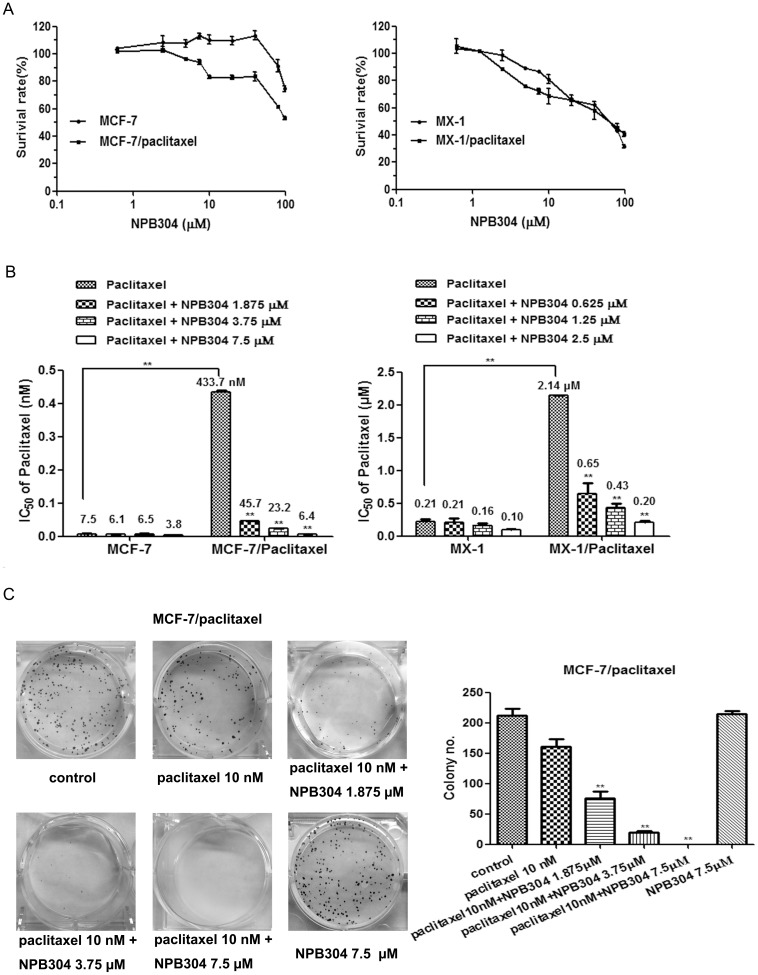
The effect of NPB304 on the paclitaxel sensitivity of resistant cells. (A) Cytotoxicity of NPB304 in the two pairs of cell lines (MX-1, MX-1/paclitaxel; MCF-7 and MCF-7/paclitaxel). (B) NPB304 reduces the IC_50_ of paclitaxel in resistant breast cancer cells. Resistant cells were treated with the indicated drugs for 72 h and subjected to an MTT assay. (C) The cells were treated with paclitaxel in the presence or absence of NPB304 for 12 days. Colony numbers were counted after Giemsa staining. *p<0.05, **p<0.01, Student's t-test (n = 3) or one-way ANOVA (n = 3).

The cytotoxicity of NPB304 in two pairs of cell lines was determined by MTT assay (Fig. 2A). The concentration that allowed a cell survival rate of more than 90% was chosen. Based on the cytotoxicity curves, NPB304 was used at maximum concentrations of 2.5 µM for MX-1 and MX-1/paclitaxel cells, and 7.5 µM MCF-7 and MCF-7/paclitaxel cells, respectively.

The IC_50_ values of paclitaxel in resistant and parental cells were investigated. MX-1/paclitaxel and MCF-7/paclitaxel cells displayed 10.1-fold and 57.8-fold greater resistance, respectively, compared to parental cells (Fig. 2B). As shown in Fig. 2B, treatment with NPB304 significantly decreased the IC_50_ of paclitaxel in the two resistant breast cancer cell lines in a concentration-dependent manner. Specifically, treatment with 0.625, 1.25 and 2.5 µM NPB304 reduced the IC_50_ of paclitaxel by 3.3-, 4.9- and 10.5-fold, respectively, in MX-1/paclitaxel cells. The IC_50_ of paclitaxel was decreased 9.5-, 18.7- and 67.7-fold after combination treatment with 1.875, 3.75 and 7.5 µM NPB304, respectively, in MCF-7/paclitaxel cells. However, NPB304 had little effect on non-resistant cells, as 2.5 µM NPB304 enhanced the sensitivity of paclitaxel by 2.1-fold in MX-1 cells and 7.5 µM NPB304 enhanced the sensitivity of paclitaxel by 2.0-fold in MCF-7 cells.

We measured cell viability using colony formation assays. Significant inhibition of cell colony formation was observed with paclitaxel in combination with NPB304 in a dose-dependent manner. After treatment with 7.5 µM NPB304, paclitaxel completely inhibited colony formation. No effect was observed with NPB304 alone (Fig. 2C).

### NPB304 treatment promoted paclitaxel-induced apoptosis in resistant MCF-7/paclitaxel cells

**Figure 3:**
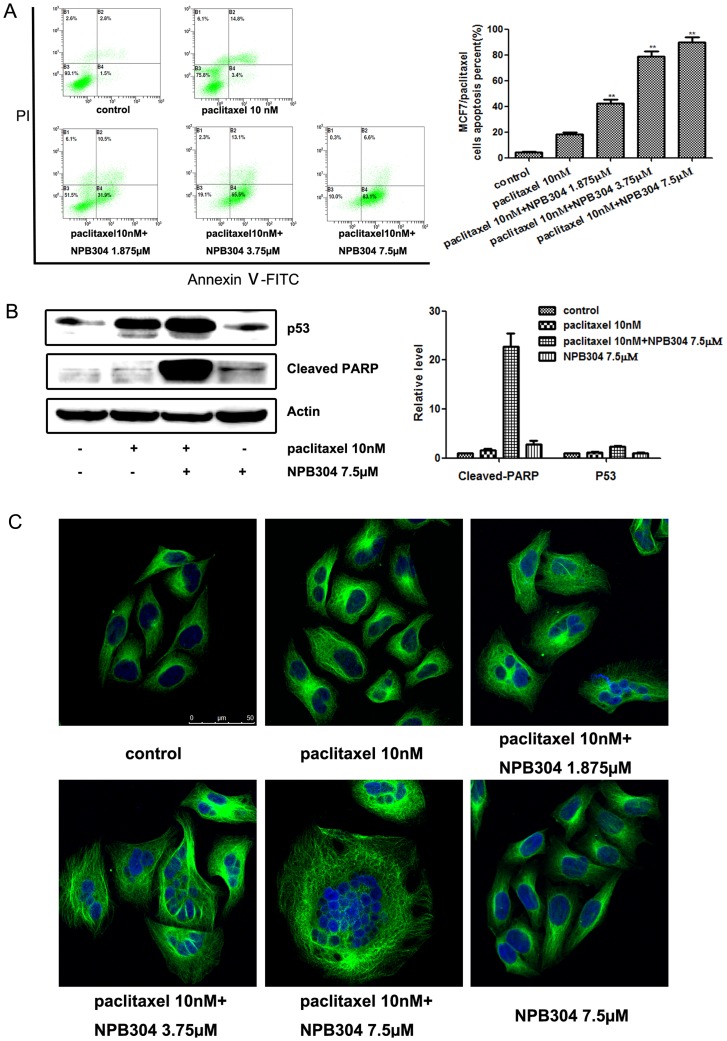
NPB304 treatment promoted paclitaxel-induced apoptosis in MCF-7/paclitaxel cells. (A) After treatment with the indicated compounds for 72 h, the cells were stained with PI and annexin V-FITC and subjected to flow cytometry analyses. (B) MCF-7/paclitaxel cells were subjected to Western blotting to measure cleaved PARP and p53 levels after 72 h of treatment. Relative protein levels were quantified by densitometry and are shown in the histogram. The experiments were performed three times. The data represent the mean ± SD. *p<0.05, **p<0.01, one-way ANOVA (n = 3). (C) NPB304 promoted the microtubule polymerization and nuclear fragmentation induced by paclitaxel. After treatment with paclitaxel with or without NPB304 for 72 h, the cells were incubated with an anti-α-tubulin antibody followed by a FITC-conjugated secondary antibody and stained with DAPI. Images were overlaid electronically after detection by confocal microscopy. Bar = 50 µm.

Consistent with its ability to inhibit growth, NPB304 increased apoptosis induced by paclitaxel (Fig. 3A). To further confirm these results, we examined some well-established biochemical markers of arrest and apoptosis: p53 and cleaved PARP. p53 regulates many genes involved in antiproliferative mechanisms, such as apoptosis and cell cycle arrest [29], [30]. Indeed, paclitaxel combined with NPB304 upregulated p53 expression and increased the level of cleaved PARP (Fig. 3B). Promoting microtubule polymerization is the main mode of action of paclitaxel. We therefore tested whether NPB304 had an effect on microtubule polymerization using an immunofluorescence assay with visualization by confocal microscopy. We observed that NPB304 combined with 10 nM paclitaxel inhibited microtubule depolymerization, promoted microtubule bundle formation and resulted in mitotic arrest (Fig. 3C); however, this result was not observed with NPB304 alone. Moreover, nuclear fragmentation was induced when the drugs were used in combination but not when paclitaxel was used alone. These results indicated that NPB304 restored the sensitivity of MCF-7/paclitaxel cells to paclitaxel in a concentration-dependent manner.

### The combination of NPB304 with paclitaxel enhanced the efficacy of paclitaxel in a xenograft model of resistant human breast cancer (MX-1/paclitaxel)

A xenograft model of resistant human breast cancer established *in vivo*, MX-1/paclitaxel, was used to evaluate the efficacy of NPB304. We were interested in exploring the sensitizing effect of different NPB304 treatment protocols on the anti-tumor efficacy of paclitaxel. The control animals received vehicle, and paclitaxel was given by i.p. injection every 3 days (15 mg/kg per injection, q3D×4). Two schedules were designed for NPB304 treatment: in the first, animals were treated with 100 mg/kg NPB304 followed 1 h later by paclitaxel (100 mg/kg, q3D×4), whereas in the second, NPB304 was administered twice daily in two separate doses (75 mg/kg or 150 mg/kg, BID) in combination with paclitaxel until the termination of the experiment. The relative tumor volume (RTV) is the ratio between the tumor volume measured at each time point and the initial tumor volume. T/C% is the ratio between the RTV of the treatment group and the RTV of the vehicle group.

**Figure 4:**
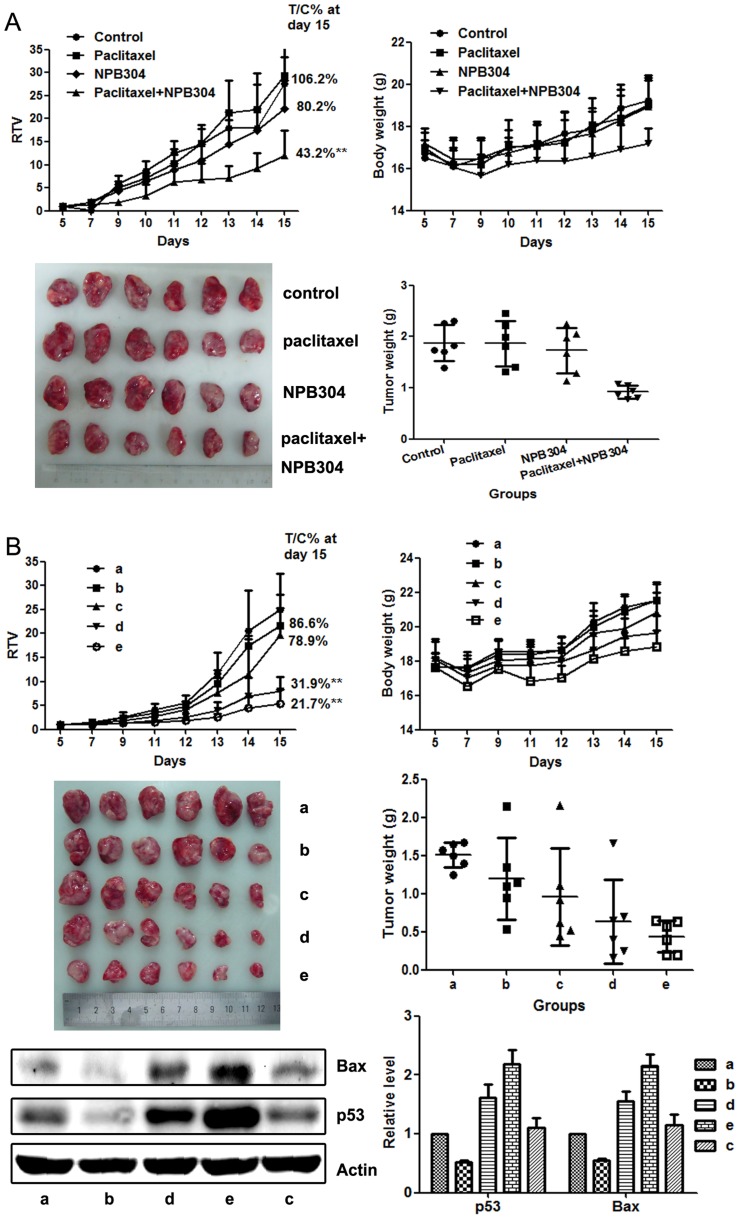
NPB304 enhanced the efficacy of paclitaxel in resistant human breast cancer xenografts (MX-1/paclitaxel). (A) The combination of NPB304 with paclitaxel in BALB/c nude mice according to the schedule one. The animals were randomized into four groups: (a) vehicle control; (b) 15 mg/kg paclitaxel (q3D×4, i.p.); (c) 100 mg/kg NPB304; (d) 100 mg/kg NPB304 (1 h before paclitaxel, p.o.) combined with 15 mg/kg paclitaxel (q3D×4, i.p.). The changes in RTV and body weight were described. The data shown are the means ± SD for each group of six mice. *p<0.05, **p<0.01, one-way ANOVA (n = 3). Fifteen days later, the mice were sacrificed, and individual tumors were weighed and photographed. (B) The administration of NPB304 with paclitaxel according to the schedule two. The animals were distributed into five groups: (a) vehicle control; (b) 15 mg/kg paclitaxel (q3D×4, i.p.); (c) 150 mg/kg NPB304 (BID); (d) 75 mg/kg NPB304 (BID) combined with 15 mg/kg paclitaxel (q3D×4, i.p.); (e) 150 mg/kg NPB304 (BID) combined with 15 mg/kg paclitaxel (q3D×4, i.p.). The changes in RTV and body weight were described. The data shown are the means ± SD for each group of six mice. *p<0.05, **p<0.01, one-way ANOVA (n = 3). Fifteen days later, the mice were sacrificed, and individual tumors were weighed and photographed. Four tumors of equal weight from five groups of nude mice were harvested for Western blotting to measure Bax and p53 levels. Relative protein levels were quantified by densitometry and are shown in the histogram.

Using the first schedule, the T/C% and the tumor weight inhibition rate were 106.2% and 0.45%, respectively, in the paclitaxel group, indicating that this model was resistant to paclitaxel *in vivo*. However, co-administration led to a significant inhibition of tumor growth compared with the vehicle or paclitaxel group, although no obvious inhibition was observed with NPB304 alone (the T/C% and the inhibition rate were 80.2% and 7.6%, respectively). The T/C% and the tumor weight inhibition rate were 43.2% and 50.9%, respectively, after combination with 100 mg/kg NPB304 (Fig. 4A). The body weight of the combination treatment groups was not significantly altered.

Using the second schedule, the T/C% and the tumor weight inhibition rate were 86.6% and 20.3%, respectively, in the group treated with paclitaxel alone; the corresponding values in the group treated with NPB304 (150 mg/kg, BID) alone were 78.9% and 35.9%, respectively. When paclitaxel was combined with 75 mg/kg NPB304 (BID), the T/C% and the inhibition rate were 31.9% and 58.1%, respectively; when paclitaxel was combined with 150 mg/kg NPB304 (BID), the corresponding values were 21.7% and 71.0%, respectively (Fig. 4B). None of the animals died, and the body weight was reduced slightly in the combination groups. In addition, we observed that the expression of apoptosis-inducing proteins, such as Bax and p53, was increased significantly in the combination group compared with the group treated with paclitaxel alone.

### NPB304 inhibits the MAPK pathway in MCF-7/paclitaxel cells

**Figure 5:**
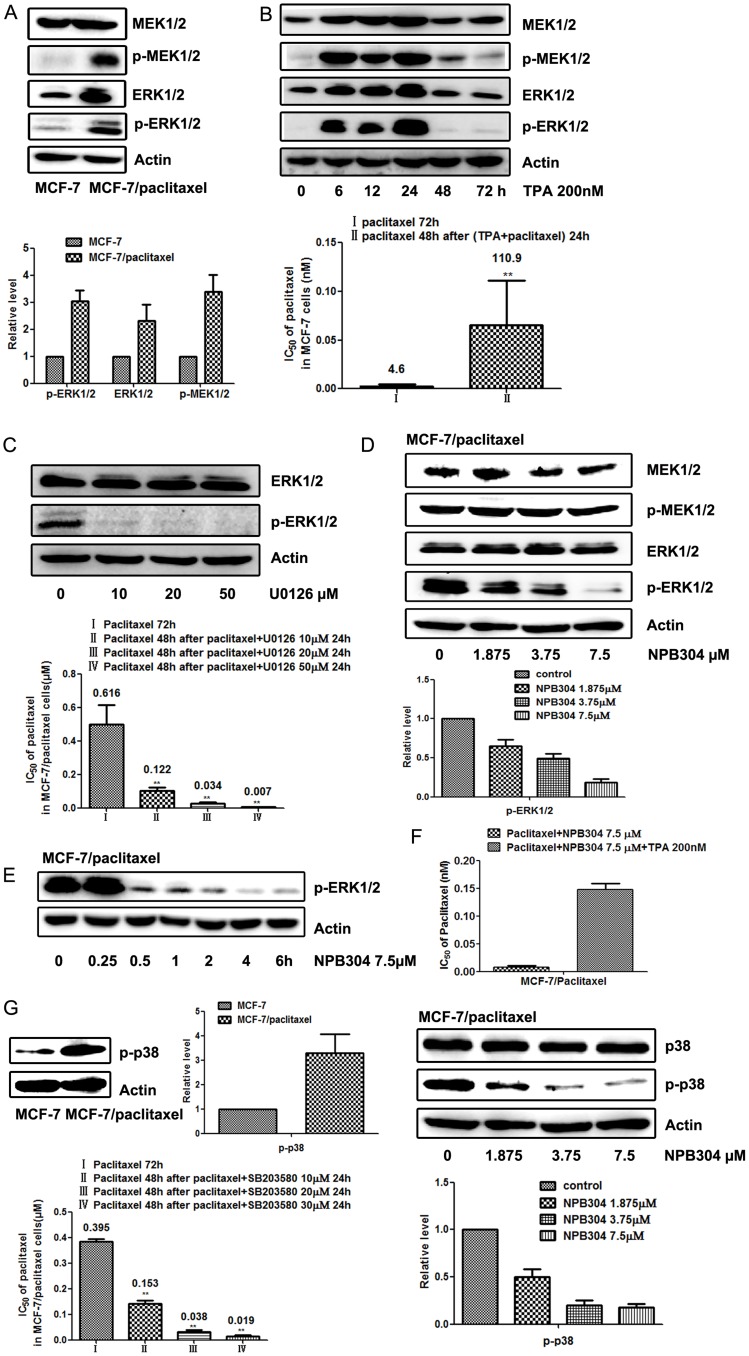
NPB304 inhibited the MAPK pathway in MCF-7/paclitaxel cells. Cell lysates were subjected to Western blotting. Relative protein levels were quantified by densitometry. (A) The MEK-ERK pathway was activated in MCF-7/paclitaxel cells compared with MCF-7 cells. (B) After treatment with 200 nM TPA (PMA) for 6 to 24 h, the MEK-ERK pathway was activated in MCF-7 cells, and p-ERK1/2 was overexpressed maximally at 24 h. MCF-7 cells were pretreated with paclitaxel and 200 nM TPA for 24 h and subsequently exposed to paclitaxel for 48 h. (C) The expression of p-ERK1/2 was decreased in a concentration-dependent manner when MCF-7/paclitaxel cells were treated with 10 to 50 µM U0126 for 24 h. Then, MCF-7/paclitaxel cells were treated with paclitaxel for 48 h after being exposed to paclitaxel and U0126 for 24 h. (D) NPB304 inhibited p-ERK in a dose-dependent manner in MCF-7/paclitaxel cells after treatment for 72 h. (E) MCF-7/paclitaxel cells were treated with 7.5 µM NPB304 for different time periods. (F) TPA partially rescued the sensitization effect of NPB304 in the MCF-7/paclitaxel cells. The resistant cells were treated with paclitaxel and NPB304 in the presence or absence of TPA (200 nM) for 72 h. (G) p-p38 was also overexpressed in MCF-7/paclitaxel cells compared with MCF-7 cells. The IC_50_ of paclitaxel was decreased when MCF-7/paclitaxel cells were treated with paclitaxel for 48 h after being exposed to paclitaxel and the p38 inhibitor SB203580 for 24 h. NPB304 suppressed the expression of p-p38 in a dose-dependent manner in MCF-7/paclitaxel cells after treatment for 72 h. The expression is shown relative to actin. The experiments were performed three times. The data represent the mean ± SD. *p<0.05, **p<0.01, one-way ANOVA (n = 3).

Antitumor drugs are known to activate the MAPK pathway and consequently induce tumor cell resistance to chemotherapy drugs [31]. We observed constitutive activation of the MAPK/ERK pathway in MCF-7/paclitaxel breast cancer cells compared with MCF-7 cells (Fig. 5A). To determine whether ERK activation causes paclitaxel resistance, we confirmed the relationship between the ERK pathway and resistance in parental MCF-7 and MCF-7/paclitaxel cells. As shown in Fig. 5B, TPA (200 nM)-induced ERK activation increased paclitaxel resistance by approximately 24-fold in MCF-7 cells. Meanwhile, U0126, an inhibitor of the MAPK/ERK pathway [32], gradually blocked ERK1/2 phosphorylation at non-toxic doses of 10, 20 and 50 µM for 24 h and increased the sensitivity of MCF-7/paclitaxel cells to paclitaxel by 5-, 18- and 89-fold, respectively, compared with paclitaxel alone (Fig. 5C). MCF-7/paclitaxel cell viability was greater than 93% when the cells were treated with 10, 20 or 50 µM U0126 alone. The above data verified the role of MAPK/ERK signaling in paclitaxel resistance in MCF-7/paclitaxel cells. We therefore tested the effect of NPB304 on MAPK/ERK signaling. As expected, NPB304 decreased ERK1/2 phosphorylation in a dose- and time-dependent manner in MCF-7/paclitaxel cells, but not in parental cells (Fig. S1), which is consistent with its ability to reduce the IC_50_ of paclitaxel in MCF-7/paclitaxel cells (Fig. 5D and 5E). Moreover, the ERK activator TPA partially rescued the sensitization effect of NPB304 to paclitaxel in the MCF-7/paclitaxel cells. Specifically, the IC_50_ of paclitaxel in the MCF-7/paclitaxel cells combined with NPB304 increased by 15-fold in the presence of TPA compared with that in the absence of TPA (Fig. 5F). Taken together, these data indicate that NPB304 inhibited the MAPK/ERK pathway via p-ERK1/2 suppression to sensitize resistant cells during paclitaxel exposure.

MCF-7/paclitaxel cells are also characterized by increased levels of MAPK/p38 compared with parental MCF-7 cells (Fig. 5G). MAPK/p38 inhibition in combination with antitumor drugs could represent a strategy for overcoming chemoresistance [33]–[35]. In this study, the p38 inhibitor SB203580 at 10, 20 and 30 µM concentrations sensitized MCF-7/paclitaxel cells to paclitaxel by 2.6-, 10.5- and 20.5-fold, respectively, compared with paclitaxel alone. Simultaneously, we observed that the expression of p-p38 was suppressed in a dose-dependent manner in MCF-7/paclitaxel cells when treated with NPB304 for 72 h. Thus, we concluded that NPB304 restored the sensitivity of resistant cells also by inhibiting the MAPK/p38 pathway.

### NPB304 increased paclitaxel accumulation by affecting the function and activity of P-gp

**Figure 6:**
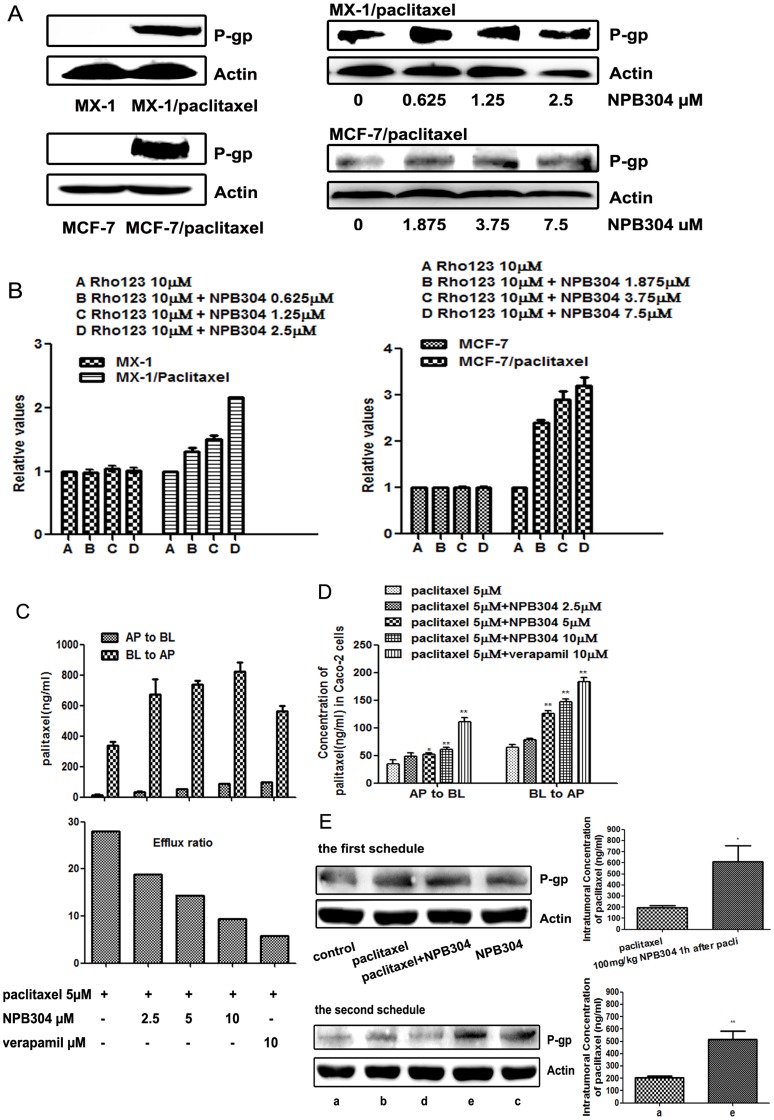
NPB304 affected P-gp function and increased paclitaxel accumulation in P-gp-overexpressing resistant cells and tumor tissue. (A) P-gp was overexpressed in resistant breast cancer cells compared with parent cells. NPB304 treatment had no effect on P-gp expression. (B) NPB304 treatment increased Rh123 accumulation in MX-1/paclitaxel cells and MCF-7/paclitaxel cells. Resistant cells were treated with the indicated drugs for 3 h, and 10 µM Rh123 was subsequently added and incubated for an additional 30 min. The mean fluorescence intensity of intracellular Rh123 was determined by flow cytometry. Cells that were not exposed to NPB304 served as negative controls. The relative value was the ratio of the mean fluorescence intensity of intracellular Rh123 of the groups and that of the negative controls. The data represent the mean ± SD. *p<0.05, **p<0.01. (C) NPB304 treatment promoted bidirectional permeability but decreased the efflux ratio of paclitaxel in a Caco-2 monolayer model. Caco-2 monolayers were treated with paclitaxel (5 µM) alone and paclitaxel combined with NPB304 or Vrp on both sides. The concentration of paclitaxel on the opposite side was determined at 3 h. The data represent the mean ± SD. The efflux ratio was the ratio of BL to AP and AP to BL. (D) NPB304 increased the intracellular concentration of paclitaxel on both sides. The Caco-2 cells were harvested to determine the intracellular concentration of paclitaxel using LC-MS-MS after 3 h. (E) NPB304 increased the intratumoral concentration of paclitaxel without reducing the P-gp expression. Four nude mice tumors with equal weights were harvested from the treatment groups to detect the expression of P-gp by Western blotting and the intratumoral concentration of paclitaxel using an LC-MS/MS assay. *p<0.05, **p<0.01.

We found that P-gp was overexpressed in both resistant breast cancer cell lines compared with parental cells (Fig. 6A) [27]. However, NPB304 had no significant effect on P-gp expression in either MX-1/paclitaxel or MCF-7/paclitaxel cells. To determine whether NPB304 affected P-gp function, intracellular accumulation of the P-gp substrate Rh123 was determined. The mean Rh123 fluorescence intensity was 55.4 in MX-1 cells, 22.1 in MX-1/paclitaxel cells, 214 in MCF-7 cells and 39.4 in MCF-7/paclitaxel cells, which demonstrated that P-gp serves as an efflux transporter in resistant cells. Rh123 accumulation was increased by 1.35-, 1.55- and 2.16-fold in MX-1/paclitaxel cells in the presence of 0.625, 1.25 and 2.5 µM NPB304, respectively. Meanwhile, Rh123 accumulation increased by 2.36-, 2.86- and 3.17-fold in MCF-7/paclitaxel cells treated with 1.875, 3.75 and 7.5 µM NPB304, respectively (Fig. 6B). NPB304 had very modest effect on the efflux activity in the parent MX-1 and MCF-7 cells, in agreement with the above results of P-gp expression and cell experiments. Taken together, these results suggest that NPB304 might inhibit the transport function and activity of P-gp in resistant breast cancer cells.

To further confirm the effect of NPB304 on P-gp function, we determined the concentration of the P-gp substrate paclitaxel in the presence or absence of NPB304 using a Caco-2 monolayer model. Three hours after administration, the concentration of paclitaxel was increased on both sides in the presence of NPB304; however, the efflux ratio (the ratio between the paclitaxel concentration from the BL to the AP side and that from the AP to the BL side) was decreased in an NPB304 concentration-dependent manner (1.5-, 1.9- and 3-fold at concentration of 2.5, 5 and 10 µM). However, the effect of NPB304 was weaker than that of Vrp (4.8-fold) (Fig. 6C). To determine the level of paclitaxel accumulation in Caco-2 cells, the cells were harvested and assayed. We found that the intracellular concentration of paclitaxel was increased in the presence of NPB304, regardless of whether it was present at the basolateral or apical side (Fig. 6D). These results were in agreement with the notion that NPB304 increased paclitaxel accumulation in resistant P-gp-overexpressing cells by affecting P-gp function. In summary, NPB304 treatment promoted bidirectional permeability in a Caco-2 transport model and increased the intracellular concentration of paclitaxel in P-gp-overexpressing Caco-2 cells.

NPB304 increased the concentration of paclitaxel in tumor tissue as well without reducing the P-gp expression. Animals were sacrificed after 15 days, and tumor xenografts were harvested to determine the paclitaxel concentration using an LC-MS/MS assay. Using the first schedule, we found that treatment with 100 mg/kg NPB304 increased paclitaxel accumulation by 3.1-fold in tumors compared to treatment with paclitaxel alone (Fig. 6E). Using the second schedule, the intratumoral concentration of paclitaxel was increased by 2.5-fold in the combination group (150 mg/kg NPB304, BID) compared with the group treated with paclitaxel alone, as determined using an LC-MS/MS assay.

## Discussion and Conclusion

Traditional cytotoxic agents remain the cornerstone of many therapies, and modulating resistance is still an important way to increase sensitivity to chemotherapy drugs such as paclitaxel. Resistance modulators that target efflux pumps have not yet found a role in oncologic practice due to their limitations, such as pharmacokinetic interactions and enhanced toxicity at non-target sites [19], [36]. In this study, we developed a novel modulator, NPB304, with a new structure and found that it enhanced the therapeutic efficacy of paclitaxel *in vitro* and *in vivo*. Furthermore, the results indicated that NPB304 increased the sensitivity of resistant cells through more than one mechanism, including inhibition of the MAPK pathway and increasing paclitaxel accumulation.

Several taxane-based MDR reversal agents have been reported in recent years [37], [38]. Based on previous research regarding taxane derivatives that was conducted at our institute [22], [24], we developed hundreds of compounds based on SIA because these derivatives are easy to synthesize and have good bioavailability. NPB304 was selected from a screen of these novel compounds because of its optimal structure, high efficiency and low toxicity *in vitro* and *in vivo* in preliminary experiments. The structural requirements for hydrophobic modifiers and the positional preference of NPB304 have been investigated in our structure-activity relationship (SAR) study (data not shown).

Previously, we investigated the potential of NPB304 to sensitize resistant breast cancer cells to paclitaxel. The chosen concentration was non-toxic or weakly toxic (inhibition rate <12%) in *in vitro* experiments (Fig. 2A). We evaluated the efficacy of NPB304 in resistant cell lines, including triple-negative MX-1 breast cancer cells and estrogen-dependent MCF-7 breast cancer cells. As expected, NPB304 displayed sensitizing activity in both resistant breast cancer cell lines (Fig. 2B and 2C). Paclitaxel in combination with NPB304 enhanced a modest degree of tubulin polymerization compared with paclitaxel alone; however, the difference was not significant (Fig. S2). This finding may help to explain why NPB304 modestly increased the efficacy of paclitaxel in parental cells (Fig. 2B).

Consistent with the results of MTT assays, treatment with NPB304 at non-cytotoxic concentrations promoted cell apoptosis induced by paclitaxel (Fig. 3A). NPB304 enhanced paclitaxel-induced apoptosis in a p53-dependent manner [39], [40] and increased PARP cleavage (Fig. 3B). Paclitaxel is a microtubule-stabilizing agent; thus, it exerts its activity mainly by promoting microtubule polymerization, inhibiting cell mitosis and inducing apoptosis [41]. Based on confocal experiments, we found that treatment with NPB304 led to paclitaxel-induced microtubule polymerization and nuclear fragmentation in a dose-dependent manner (Fig. 3C).

NPB304 also increased the paclitaxel sensitivity of MX-1/paclitaxel triple-negative breast cancer xenografts in nude mice [13], [14], [42]. Because the two schedules were administered separately and we did not use MX-1/paclitaxel cells of the same passage in the xenografts, there were some differences in the inhibition rates of paclitaxel. However, the inhibition rates were controlled in the range of 0–20% to ensure that the model was resistant to paclitaxel (Fig. 4). This reversal of activity *in vivo* in preclinical studies requires further validation in other human tumor xenografts to confirm the effects.

Because the MAPK pathway is generally associated with cell proliferation and survival, it has been considered to be an attractive target for anticancer therapies [43]–[45]. Previous studies have demonstrated that activation of the MAPK pathway can increase tumor resistance [46], [47]. Anticancer agents such as paclitaxel are known to activate the MAPK/ERK pathway, which leads to drug resistance in cancer cells [31]. Previous studies have also indicated that MAPK/ERK pathway inhibition enhanced paclitaxel-induced apoptosis [11], [48]–[51]. In this study, we observed constitutive activation of the MAPK/ERK pathway in MCF-7/paclitaxel cells, which was not observed in MCF-7 cells (Fig. 5A). However, the effect of MAPK inhibition on paclitaxel-induced apoptosis may be dependent on the genetic/epigenetic status of the cell type and the degree to which cells undergo paclitaxel-induced ERK activation [52]. Therefore, we subsequently identified the relationship between the activated MAPK/ERK pathway and resistance in MCF-7/paclitaxel cells. We found that activation of the MAPK/ERK pathway with TPA decreased paclitaxel sensitivity in MCF-7 cells (Fig. 5B), supporting the role of this pathway in paclitaxel resistance. In agreement with a previous report [7], sustained activation of the ERK pathway resulted in resistance to paclitaxel in MCF-7/paclitaxel cells. On the other hand, our findings demonstrate that blocking the ERK pathway with U0126 in ERK-activated MCF-7/paclitaxel cells restored paclitaxel sensitivity (Fig. 5C), suggesting that the ERK pathway may play an important role in breast cancer cell resistance. Interestingly, we determined that NPB304 interfered with the MAPK/ERK pathway and inhibited p-ERK1/2 (Fig. 5D and 5E). And the activation of ERK with TPA partially blocked the reversal effect of NPB304 in the MCF-7/paclitaxel cells. Moreover, some studies have reported that inhibition of the MAPK/p38 pathway significantly decreased chemotherapy drug resistance in resistant cells [53]. In this study, we observed that the IC_50_ of paclitaxel decreased in the presence of the p38 inhibitor SB203580 in MCF-7/paclitaxel cells, and we found that NPB304 downregulated the expression of p-p38 in MCF-7/paclitaxel cells with MAPK/p38 activation (Fig. 5G). Taking these findings together, we concluded that NPB304 increased the sensitivity of resistant cells through inhibition of the MAPK pathway.

Co-administration of NPB304 with paclitaxel increased paclitaxel accumulation in tumor tissue [27] and Caco-2 cells, both of which overexpress P-gp. Overexpression of P-gp was identified as the main factor mediating MDR in various types of resistant cancer [54], [55]; thus, we examined the effect of NPB304 on the transport function and expression of P-gp. NPB304 increased accumulation of the P-gp substrate Rh123 in both resistant cell lines; however, this result was not achieved via downregulation of P-gp expression. Similarly, NPB304 enhanced bidirectional paclitaxel permeability but reduced the efflux ratio. In summary, we deduced that NPB304 enhanced paclitaxel accumulation by affecting the function and activity of P-gp.

Previous work has shown that components of the MAPK pathway, especially p-p38, play an important role in P-gp activity [56]. Moreover, inhibition of p38 (and ERK) may inhibit P-gp activity and attenuate MDR [57], [58]. In this study, NPB304 interfered with the expression of p-p38, simultaneously influencing the functional activity of P-gp. Therefore, we speculated that NPB304 reduced P-gp efflux activity and increased the concentration of paclitaxel, possibly by inhibiting the MAPK/p38 pathway. However, this speculation requires further investigation.

Taken together, our results demonstrate that NPB304 increased the paclitaxel sensitivity of resistant breast cancer cells by continually suppressing p-ERK1/2 and p-p38 in the MAPK pathway and inhibiting P-gp function to increase the intracellular concentration of paclitaxel. Furthermore, we confirmed the sensitizing effect of NPB304 in a tumor xenograft model, supporting the potential use of NPB304 in combination with paclitaxel in breast cancer chemotherapy to overcome multidrug resistance.

## Supporting Information

Figure S1 **The effect of NPB304 on the expression of p-ERK1/2 in parent MCF-7 cells.** When treated with the indicated concentration of NPB304 for 72 h, the expression of p-ERK1/2 did not decrease in MCF-7 cells.
(DOC)

Figure S2 **NPB304 modestly enhances tubulin polymerization induced by paclitaxel.** *In vitro* tubulin polymerization assays were conducted using the porcine tubulin- and fluorescence-based tubulin polymerization assay kit (Cat. #BK011P; Cytoskeleton, Denver, CO, USA) following the manufacturer's protocol. The data were analyzed with Softmax pro software (Ex. 340–360 nm±20 nm, Em. 410–460 nm±20 nm). NPB304 alone had almost no effect on microtubule dynamics. Paclitaxel in combination with NPB304 modestly enhanced tubulin polymerization compared with paclitaxel alone.
(DOC)
