# Supplementary material for: A New 2α,5α,10β,14β-tetraacetoxy-4(20),11-taxadiene (SIA) Derivative Overcomes Paclitaxel Resistance by Inhibiting MAPK Signaling and Increasing Paclitaxel Accumulation in Breast Cancer Cells
Source: PLoS One. 2014 Aug 5;9(8):e104317. doi: 10.1371/journal.pone.0104317 (PMC4122450; doi:10.1371/journal.pone.0104317)

**Figure S1. The effect of NPB304 on the expression of p-ERK1/2 in parent MCF-7 cells.** When treated with the indicated concentration of NPB304 for 72h, the expression of p-ERK1/2 was not decreased in MCF-7 cells.


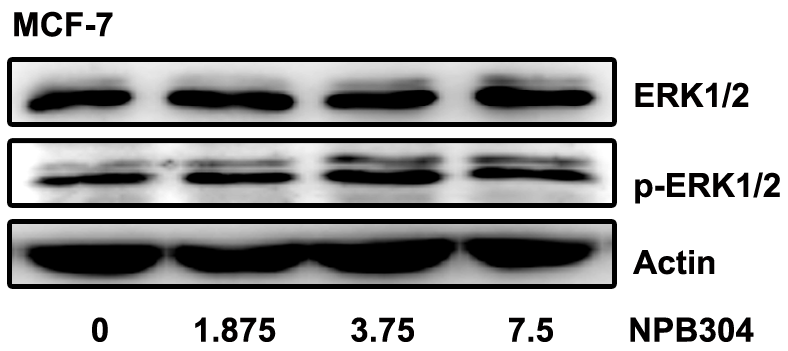

Supplement: Figure S1 — The effect of NPB304 on the expression of p-ERK1/2 in parent MCF-7 cells. When treated with the indicated concentration of NPB304 for 72 h, the expression of p-ERK1/2 did not decrease in MCF-7 cells. (DOC) [file pone.0104317.s001.doc]
