# Supplementary material for: A New 2α,5α,10β,14β-tetraacetoxy-4(20),11-taxadiene (SIA) Derivative Overcomes Paclitaxel Resistance by Inhibiting MAPK Signaling and Increasing Paclitaxel Accumulation in Breast Cancer Cells
Source: PLoS One. 2014 Aug 5;9(8):e104317. doi: 10.1371/journal.pone.0104317 (PMC4122450; doi:10.1371/journal.pone.0104317)
Supplement: Figure S2 — NPB304 modestly enhances tubulin polymerization induced by paclitaxel. In vitro tubulin polymerization assays were conducted using the porcine tubulin- and fluorescence-based tubulin polymerization assay kit (Cat. #BK011P; Cytoskeleton, Denver, CO, USA) following the manufacturer's protocol. The data were analyzed with Softmax pro software (Ex. 340–360 nm±20 nm, Em. 410–460 nm±20 nm). NPB304 alone had almost no effect on microtubule dynamics. Paclitaxel in combination with NPB304 modestly enhanced tubulin polymerization compared with paclitaxel alone. (DOC) [file pone.0104317.s002.doc]

**Figure S2. NPB304 enhances tubulin polymerization induced by paclitaxel modestly.** In vitro tubulin polymerization assays was conducted by Tubulin Polymerization Assay kit (Porcine tubulin and Fluorescence based, Cat. # BK011P) (Cytoskeleton, Acoma St. Denver, USA) following manufacture’s protocol. Data were analyzed with Softmax pro software (Ex. 340-360 nm ± 20 nm, Em. 410-460 nm ± 20 nm.). There was almost no affect on microtubule dynamics by using NPB304 itself. Combination with NPB304 enhanced a modest degree of tubulin polymerization in a concentration-dependent manner compared with paclitaxel alone.
